# The role of community-based health services in influencing postnatal care visits in the Builsa and the West Mamprusi districts in rural Ghana

**DOI:** 10.1186/s12884-018-1926-7

**Published:** 2018-07-09

**Authors:** Evelyn Sakeah, Raymond Aborigo, James Kotuah Sakeah, Maxwell Dalaba, Ernest Kanyomse, Daniel Azongo, Dominic Anaseba, Samuel Oladokun, Abraham Rexford Oduro

**Affiliations:** 1 Navrongo Health Research Centre, Post Office Box 114, Navrongo, Upper East Region, Ghana; 2 Department of Medicine, University of Calgary, Alberta, Canada

**Keywords:** Community-based health service delivery-Ghana- maternal mortality-women service utilization-postnatal care

## Abstract

**Background:**

Globally, maternal mortality is still a challenge. In Ghana, maternal morbidity and mortality rates remain high, particularly in rural areas. Postnatal Care (PNC) is one of the key strategies for improving maternal health. This study examined determinants of at least three PNC visits in rural Ghana.

**Methods:**

We conducted a cross-sectional study at the Community-Based Health Planning and Services (CHPS) Zones in the Builsa and West Mamprusi Districts between April and June 2016. We selected 650 women who delivered within 5 years preceding the survey (325 from each of the two sites) using the two-stage random sampling technique.

**Results:**

Of the 650 respondents, 62% reported attending postnatal care at least three times. In the Builsa district, the percentage of women who made at least three PNC visits were 90% compared with 35% in the West Mamprusi district. Older women and those who attended antenatal clinics at least four times (AOR: 5.23; 95% CI: 2.49–11.0) and women who had partners with some secondary education (AOR: 3.31; 95% CI: 1.17–9.39) were associated with at least three PNC visits.

**Conclusions:**

Men engagement in maternal health services and the introduction of home-based PNC services in rural communities could help health workers reach out to many mothers and children promptly and improve PNC visits in those communities.

## Background

Despite the establishment of a number of global and national initiatives to improve maternal health, maternal mortality is still a major concern in sub-Saharan Africa [1]. Every year in Africa, at least 125,000 women and 870,000 newborns die in the first week after birth [2]. In 2015, global maternal mortality was 303,000 with sub-Saharan Africa accounting for the highest number of deaths (201,000) [1]. In 2015, Ghana recorded 2800 maternal deaths which is high when compared with other sub-Saharan Africa countries [1]. Ghana is one of the countries that failed to achieve the targets set for the fourth and fifth Millennium Development Goals [3].

Postnatal care (PNC) is the care given to the mother and her newborn baby immediately after the birth and for the first 6 weeks of life [2]. The PNC period is crucial for both the mother and the newborn [2, 4, 5]. The first 6 weeks after birth is known as the PNC period [2]. The first day after birth is associated with the highest risk for both mother and baby [2]. Most deaths occur during the first 24 h after childbirth and half of maternal and neonatal deaths occur during the first week after birth [4]. Postpartum haemorrhage is the main cause of maternal mortality [4, 5]. Other causes include complications from unsafe abortion, hypertensive disorders during pregnancy, postpartum infections, obstructed labor [6] puerperal sepsis [5] and HIV [4].

For babies, many of them die due to birth asphyxia during the first day after birth and a lot more because they are born preterm [2]. In the second week after birth, about 38% of them in sub-Saharan Africa die due to infections [2]. Long term disability and poor development of both the child and the mother is reported to be associated with the early postnatal period [2].

Recognizing the role of PNC during this critical period for mothers and babies, the WHO recommended at least three PNC visits for all nursing mothers to ensure their survival and that of their newborns [7]. PNC is important to identify and manage complications that arise as a result of child birth and to provide health information that is beneficial to both mother and baby [2, 4, 5]. It ensures monitoring of women and their newborns in the pueperium so as to reduce morbidity and mortality risks. PNC also ensures prompt access to family planning services to prevent poorly spaced pregnancies [2].

In the national safe motherhood service protocol of Ghana, it is specified that all pregnant women attend PNC at least three times for uncomplicated pregnancies with more visits recommended in case of complications [8]. The first PNC visit is within 48 h, the second by day 7 and the third, 6 weeks after birth [9]. PNC is high (78%) across the country but the proportion of women receiving a PNC check-up within 2 days of delivery is higher in urban than rural settings [10]. Also, the number of women receiving the three recommended PNC visits in rural Ghana is low [10], thus necessitating an enquiry into the factors that are associated with such low attendance.

A number of studies have examined factors that influence utilization of PNC services by mothers [11–20]. Some have highlighted important linkages between demographic factors such as parity, birth rank, number of pregnancies, religion, mother’s education and utilization of PNC services [14–17, 20]. Studies in developing countries such as Ethiopia and Nepal show that access to economic resources ensures that women are able to finance their deliveries in health facilities and are able to obtain subsequent services such as PNC [15, 17]. Besides, women with better financial resources have a better chance of benefiting from health education campaigns through media outlets like television, and newspapers because they are able to afford them [17]. Also, physical access as well as the geographical location of the mother have been shown to influence the use of PNC services [15, 19]. At the individual and social levels, factors like ANC attendance, skilled attendant during child birth, place of delivery, previous use of PNC services, and experience of medical complications have had significant influence on utilization of PNC services as observed by several studies [13, 15, 16, 19].

UNICEF together with the Ghana Health Service pilot-tested a package of interventions that focused on maternal, neonatal and child health (MNCH) within the framework of the life cycle approach in the Builsa Districts from 2012 to 2017 aimed at reducing maternal, newborn and child health mortality in the Builsa Districts [21]. As part of the intervention strategies UNICEF/Ghana Health Service implemented home-based postnatal care in the Community-based Health Planning and Services (CHPS) zones. The health providers visited women, who resided far away or were unable to come to the health facilities for care to provide postnatal services at their doorsteps [21]. This study evaluates the effectiveness of the intervention program comparing PNC service access in CHPS zones in two rural communities, one with home-based postnatal care (Builsa) and one without this service (West Mamprusi).

### Primary health care

Primary health care in Ghana is delivered through the CHPS Program and the health centres. After many years of investigation into appropriate strategies for service delivery in rural settings, the CHPS Initiative has adapted strategies tested in the successful Navrongo experiment to guide national health reforms that mobilize volunteerism, resources and cultural institutions for supporting community-based primary health care [22]. In 2000 the Ghana Health Service began training Community Health Officers (CHOs) to provide basic services in rural areas through the CHPS program [22]. The CHPS initiative is a community-based health program aimed at increasing availability and access to basic health services including maternal and child health services to rural communities in Ghana [22]. It is a government initiative that brings together CHOs and community members to deliver health services to rural communities [22, 23]. The overall strategic goal of CHPS is to improve the health status of the population by strengthening the health system and empowering communities and households for service delivery and utilization [24]. This study was conducted in CHPS zones to ascertain the percentage of women who have had at least three PNC visits and determine factors associated with at least three PNC visits.

## Methods

### Study setting

The study was carried out in the Builsa and the West Mamprusi Districts of the Upper East and Northern Regions of Ghana. The population estimate for the 2010 census for the Upper East region was 1,046,545 with 79% being rural while that of the Northern region was 2,479,461 with 70% rural dwellers [25].

The Builsa district which is located in the Upper East Region of Ghana, has a population of 92,991 [25]. In 2012, the district was divided into two-Builsa North and South Districts -but for the purposes of this study, the old demarcation and name of the district would be maintained. The Builsas constitute about 83% of the entire population and the remaining 17% is made up of minor ethnic groups [25]. The district is served by a district hospital located at Sandema, the district capital, four health centres, two private clinics and twenty functional CHPS zones with resident CHOs [26].

The West Mamprusi District was carved out of the Gambaga District in 1988 under the Government of Ghana’s decentralization and local government reform policy. The district’s administrative capital is Walewale, which lies on the Tamale-Bolgatanga trunk road, approximately 68 miles away from Tamale. The West Mamprusi District has a population of 117,821 made up of mostly Mamprusis who constitute about 75% of the total population [25]. There are three main ethnic groups (Mamprusis, Kantosis and Comas) mixed with settlers such as Frafras, Kassenas, Bulisas, Zambarmas and Hausas. The most widely spoken language is Mampruli [26]. The districts’ health infrastructure is made up of one hospital in Walewale, one polyclinic, one health centre, two clinics and six functional CHPS zones with resident CHOs [26].

The study was conducted in the two districts because of the vast differences in health infrastructure, particularly the number of CHPS compounds and human resources.

### Study design

This was a cross-sectional household survey with women who had ever given birth in the 5 years prior to the study in the Builsa and the West Mamprusi Districts.

### Sample size and sampling

The sample size was calculated based on the average proportion of skilled delivery (50%) in the Upper East and Northern Regions with 5 year annual average of skilled delivery (2005–2009) of 1250 in the two districts (Builsa and West Mamprusi Districts) and 95% confidence interval as well as a corresponding *p* <  0.05 for significance. We used the formula for sample size *n* = {DEFF*Np (1-p)}/ {(d^2^/Z^2^_1-α/2_*(N-1) + p*(1-p)} [27] and this gave a sample size of 295 women. We assumed a refusal rate of 10%, which brought the total sample size for each district to 325 women.

A two-stage sampling method was used and the primary sampling unit was the enumeration area (EA), defined as the geographic area canvassed by one census representative. Sampling EAs and households was based on the assumptions that: [1] CHOs were working in these EAs in the two districts and [2] there is homogeneity in the provision of CHO services across the EAs.

The first stage involved selecting geographical clusters or EAs, from an updated master sampling frame constructed from the 2000 Ghana Population and Housing Census. A total of 50 clusters were selected from the master sampling frame of 323 (West Mamprusi-197 and Builsa Districts-144). The clusters were randomly selected from the list of EAs in each district. The selection was based on a simple random technique. In the second stage of selection, we listed all households in the selected enumeration areas (clusters) and used the simple random sampling method to sample 50 of them from each selected cluster. We compiled the list of women with children less than 5 years in each household and randomly selected one of them for the interview. For the random selection process, each eligible member of the household was assigned a unique number, then each number was placed in a bowl and mixed thoroughly. The fieldworker then randomly picked numbered tags from the bowl and interviewed the selected women. A total of 1623 eligible women were on the household list compiled for the study.

### Data collection

Data was collected using a structured questionnaire, which included items concerning social and demographic characteristics, such as age, religion, marital status, education and partner’s education, the wealth of the woman, ethnicity, religion, ANC attendance during previous pregnancy, skilled attendant at birth and PNC visits. Age represented the woman’s age at the time of interview, marital status reflected the type of union the respondents were involved in. Woman and partner’s education reflected the educational levels of respondents and their partners, respectively. Wealth index reflected their financial status, which was based on household assets. The questionnaire was developed in the English language, but the research team translated it into Buli and Mampruli-the major languages of the study areas. The questionnaire was pretested in communities outside the study sites. We trained six senior high school graduates as fieldworkers and two university graduates as supervisors for the data collection. The fieldworkers had a two-week training prior to the survey and visited households to interview eligible women. We started fieldwork on the 14th of April and ended on the 13th of June, 2016.

### Data analysis

We performed descriptive statistics, bivariate and multivariable analysis. All *p*-values were two-tailed, and a value of *p* <  0.05 was considered significant. The outcome variable for the analysis was at least three PNC visits (Yes or No). This was ascertained by the question: How many times did you receive PNC after your last child birth? We included explanatory variables such as age of the woman, marital status, geographical location, women’s education, husband’s education, wealth index, religion, ANC attendance and type of delivery. The wealth index consisted of 23 household-related items. These independent variables were selected based on previous studies [28–31]. We generated quintile ranks for wealth status using principal component analysis.

All statistical analyses were performed using Stata Version 12 (Stata Corp., TX).

## Results

### Respondents’ socio-demographic characteristics

Table 1 shows the characteristics of 650 women from the Builsa and the West Mamprusi districts that participated in the study. The number of respondents from these districts were 325 and 325 respectively. Of all the respondents, 50% were Builsa, 29% were Mamprusi, and 21% were other tribes living in the West Mamprusi district. Half of the respondents were Christians, 37% practiced Islam and 28% worshipped ancestral religion (Table 1). Slightly over half of the women and nearly three quarters of their husbands had no education. However, more of the men attained secondary or higher education than the women.

**Table 1:** Socio-demographic characteristics of respondents

Characteristics	Builsa District (*n* = 325) N (%)		West Mamprusi District (*n* = 325) N (%)		Both Districts (*n* = 650) N (%)		*p*-value^†^
Age group							< 0.001
15–24	108	(33.7)	63	(19.6)	171	(26.7)	
25–34	133	(41.6)	182	(56.7)	315	(49.1)	
35–49	79	(24.7)	76	(23.7)	115	(24.2)	
Religion							< 0.001
Traditional	70	(21.6)	13	(4.0)	83	(12.8)	
Christianity	249	(76.9)	79	(24.3)	328	(50.5)	
Islam	5	(1.5)	233	(71.7)	238	(36.7)	
Ethnicity							< 0.001
Builsa	324	(99.7)	2	(0.6)	326	(50.2)	
Mamprusi		NA	188	(57.9)	188	(28.9)	
Other	1	(0.3)	135	(41.5)	136	(20.9)	
Marital status							
Married	309	(95.4)	317	(97.5)	626	(96.5)	0.135
Single/widowed/separated	15	(4.6)	8	(2.5)	23	(3.5)	
Education							
None	165	(50.8)	175	(53.9)	340	(52.3)	
Primary	69	(21.2)	73	(22.5)	142	(21.9)	
Middle/JSS/JHS	64	(19.7)	46	(14.1)	110	(16.9)	
Secondary/SSS/SHS and above	27	(8.3)	31	(9.5)	58	(8.9)	0.305
Partner’s Education							
None	223	(68.8)	187	(57.5)	410	(63.2)	< 0.001
Primary	42	(13.0)	35	(10.8)	77	(11.9)	
Middle/JSS/JHS	31	(9.6)	36	(11.1)	67	(10.3)	
Secondary/SSS/SHS and above	28	(8.6)	67	(20.6)	95	(14.6)	
Number of children alive							
1	67	(20.6)	58	(17.9)	125	(19.2)	0.876
2	54	(16.6)	54	(16.6)	108	(16.6)	
3	64	(19.7)	70	(21.5)	134	(20.6)	
4	63	(19.4)	60	(18.5)	123	(18.9)	
5 or more	77	(20.7)	83	(25.5)	160	(20.6)	
Distance to health facility							
Less than 30 min	204	(62.8)	192	(59.1)	396	(60.9)	0.002
30 mins – 2 h	119	(36.6)	116	(35.7)	235	(36.2)	
2 h – 4 h	2	(0.6)	17	(5.2)	19	(2.9)	
Wealth Index							
Poor	40	(16.1)	117	(52.5)	157	(33.4)	< 0.001
Middle	75	(30.3)	82	(36.8)	157	(33.3)	
Rich	133	(53.6)	42	(10.7)	157	(33.3)	
ANC attendance							< 0.001
≥ 4 attendance	298	(93.1)	257	(80.8)	555	(87.0)	
≤ 3 attendance	22	(6.9)	61	(19.2)	83	(13.0)	
Skilled delivery							< 0.001
Yes	245	(75.4)	186	(57.2)	431	(66.3)	
No	80	(24.6)	139	(42.8)	219	(33.7)	
PNC visits							< 0.001
≥ 3 visits	293	(90.1)	112	(34.5)	405	(62.3)	
≤ 2 visits	32	(9.8)	213	(65.5)	245	(37.7)	

About 87% of the women reported having had at least four ANC attendance, and 66% of them were supervised by a skilled attendant during child birth and 62% had attended PNC at least three times.

The significant difference in the PNC attendance rate between the two districts indicates the need for a stratified analysis and presentation of the data.

### Factors associated with PNC visits by Study District

Table 2 presents the results of a regression analysis for at least three PNC visits according to selected characteristics, in the West Mamprusi district. The results revealed that, women who attended ANC four times (AOR: 8.88; 95% CI: 2.94–26.8), aged 36–49 (AOR: 12.33; 95% CI: 2.94–51.9), and whose partners had some secondary education (AOR: 3.66; 95% CI: 1.57–8.51) were more likely to attend PNC at least three times relative to those who attended ANC three or less times and whose partners had no education respectively.

**Table 2:** Regression Analysis Results for Postnatal Care Attendance in the West Mamprusi District

Characteristic	OR	(95% Cl)	AOR	(95% Cl)
ANC attendance				
≤ 3 attendance		**1**		**1**
≥ 4 attendance	**5.00**	**(2.19–11.41)**	**8.88**	**(2.94–26.8)**
Age group				
15–24 (r)		**1**		**1**
25–35	0.81	(0.44–1.48)	2.54	(0**.**84–7.66)
36–49	1.36	(0.68–2.70)	**12.3**	**(2.94–51.9)**
Woman’s education				
None (r)		**1**		**1**
Primary	0.97	(0.44–2.16)	1.45	(0.39–5.36)
Some secondary	1.40	(0.55–3.58)	2.95	(0.76–11.5)
Husband’s education				
None (r)		**1**		**1**
Primary	1.58	(0.74–3.36)	2.82	(0**.**89–8.95)
Some secondary	**2.33**	**(1.41–3.85)**	**3.66**	**(1.57–8.51)**
Religion				
Traditional		**1**		**1**
Christianity	3.74	(0.78–18-0)	8.03	(0.70–92.9)
Islam	2.77	(0.60–12.0)	10.5	(0.92–12.1)
Marital status				
Divorced/widowed/never married (r)		**1**		**1**
Married	0.87	(0.20–3.72)	1.01	(0.09–11.6)
Number of children alive				
1 (r)		**1**		**1**
2 + 3	0.84	(0.44–1.60)	0.60	(0.19–1.86)
4+	0.83	(0.44–1.55)	0.76	(0.20–2.86)
Distance to health facility				
Less than 30 min		**1**		**1**
30 mins – 2 h	1.19	(0.75–1.89)	0.75	(0.36–1.57)
Wealth Index				
Poor (r)		**1**		**1**
Middle	**0.55**	**(0.31–0.99)**	0.63	(0.30–1.31)
Rich	0.56	(0.22–1.42)	1.01	(0.29–3.54)
Skilled delivery				
No		**1**		**1**
Yes	1.05	(0.66–1.67)	1.31	(0.65–2.65)

Bold values are significant (*p < 0.05; **p < 0.01; ***p < 0.001) AOR: adjusted odds ratio; CI: confidence interval; JHS: junior high school; JSS: junior secondary school; OR: odds ratio; SHS: senior high school; SSS: senior secondary school

Table 3 presents regression analysis for at least three PNC visits by selected characteristics in the Builsa district. Adjusted odd ratios revealed that ANC attendance, age, women’s education, religion, marital status, partner’s education, parity, and distance to health facility, wealth and type of delivery were not associated with PNC visits.

**Table 3:** Regression Analysis Results for Postnatal Care Attendance in the Builsa District

Characteristic	OR	(95% Cl)	AOR	(95% Cl)
ANC attendance				
≤ 3 attendance		**1**		**1**
≥ 4 attendance	1.46	(0.41–5.25)	3.01	(0.64–14.2)
Age group				
15–24 (r)		**1**		**1**
25–35	1.93	(0.85–4.35)	1.44	(0.42–4.90)
36–49	2.57	(0.90–7.35)	1.24	(0.24–6.33)
Woman’s Education				
None (r)		**1**		**1**
Primary	**4.00**	**(1.34–12.0)**	2.84	(0.55–14.6)
Some secondary	2.33	(0.70–7.02)	2.35	(0.46–12.1)
Husband’s Education				
None (r)		**1**		**1**
Primary	0.57	(0.19–1.65)	0.74	(0.20–2.75)
Some Secondary	**0.34**	**(0.15–0.77)**	0.60	(0.18–2.02)
Religion				
Traditional		**1**		**1**
Christianity	1.04	(0.43–2.53)	1.51	(0.46–4.95)
Marital status				
Divorced/widowed/never married (r)		**1**		**1**
Married	0.64	(0.08–5.04)	0.45	(0.04–5.26)
Number of children alive				
1 (r)		**1**		**1**
2 + 3	1.42	(0.58–3.43)	1.01	(0.27–3.76)
4+	2.55	(0.98–6.62)	1.14	(0.21–6.16)
Distance to health facility				
Less than 30 min		**1**		**1**
30 mins – 2 h	1.88	(0.82–4.33)	1.65	(0.61–4.50)
Wealth Index				
Poor (r)		**1**		**1**
Middle	2.01	(0.54–7.37)	1.22	(0.29–5.14)
Rich	1.04	(0.36–3.05)	0.59	(0.16–2.16)
Skilled delivery				
No		**1**		**1**
Yes	0.84	(0.35–2.03)	0.85	(0.26–2.73)

Bold values are significant (*p < 0.05; **p < 0.01; ***p < 0.001)

## Discussion

The study revealed that 62% of women made at least three PNC visits, despite the wide difference between the two sites. Women attendance of four or more ANCs, age and partner’s education were associated with at least three PNC visits in the West Mamprusi district.

In the Builsa district, the percentage of women who made at least four ANC attendance, skilled delivery and at least three PNC visits were 90, 75 and 90% respectively. On the other hand, in the West Mamprusi district, the percentage of women having four ANC attendances, skilled delivery and at least three PNC visits were 81, 57 and 35% respectively. However, our results showed that the differences in use of ANC and skilled delivery in the West Mamprusi District compared to that of Builsa are not as low as that for PNC. The community engagement component of the CHPS program ensures that community health volunteers are able to identify all pregnant women and nursing mothers within their locations and monitor their use of health services [23]. This could have contributed to improved utilization of PNC services in the Builsa district. In addition, the introduction of home-based postnatal care in the Builsa district helped health workers to reach out to many mothers and children promptly and that improved PNC visits in those communities [21]. Home-based care is an integral part of health service delivery, particularly in the Builsa district. The relevance of this approach to health care delivery lies in the wide coverage of services to both mothers and children in the comfort of their homes. The approach brings health services to the doorsteps of the people and might have impacted positively on maternal health outcomes in the Builsa district (32). Thus, the home-based postnatal care strategy could have contributed to the difference in the PNC uptake between the two districts. This observation suggests that where health services are brought closer to the people, it could improve the uptake of the services including PNC as reported in other studies in Ethiopia and Tanzania [15, 32, 33].

Women who attended ANC at least four times were more likely to make three or more PNC visits. ANC offers women the opportunity to access health information and to appreciate the importance of PNC. In addition, women who make at least four ANC attendances are more likely to be those who adhere to health recommendations and therefore would make the required number of PNC visits. Evidence from other studies show that ANC attendance strongly predicts utilization of PNC services [17, 32–35].

Older women were associated with at least three PNC visits in the West Mamprusi district. These findings contradict those of Workineh and Hailu (2014), Neupane and Doku (2012) and Rwabufigiri et al. (2016) who said that women with multiple pregnancies usually perceive themselves as having acquired enough experience to handle subsequent pregnancies, hence they fail to use PNC services [20, 36, 37]. The long interaction between women and CHOs through the CHPS program and other community engagement strategies [23, 38] could have contributed to the older women knowing the importance of PNC and therefore utilizing the services. The findings suggest that, previous reproductive health activities such as ANC, skilled delivery, PNC and family planning services offered an important avenue for older women to establish contact with health services and develop a good relationship with health care providers which ultimately leads to an increase in utilization. Also, past experience of medical complications could have influenced the use of PNC services by older women as observed in other studies [15, 16, 19].

Higher educational level of partner was positively associated with at least three PNC visits in the West Mamprusi district. In northern Ghana, men as heads of their lineages have power and authority to make decisions pertaining to their families including maternal health care [39–42]. Although research has shown men’s involvement in maternal health services [43, 44], they could do more to improve the health seeking behaviour of their partners by reminding them about appointment days, encouraging them to seek skilled care and accompanying them to the health facility for maternal health services. Also, in patriarchal societies where women lack autonomy, their partners should be given information on the need for routine visits to health care providers during pregnancy as well as the postpartum period to improve maternal and newborn survival.

### Study limitations

This study had some limitations. First, recall bias was a potential limitation because some participants could have forgotten about past events involving PNC services, but to minimize this bias, past events were limited to the 5 years preceding the interview. Using different local languages to collect the data could also have distorted the presentation of the questions to the respondents. However, the standard training for fieldworkers and supervisors and the in-depth translation and back translation of the questions minimized the language bias.

## Conclusions

Women who have had four ANC visits and those whose partners received some secondary education are more likely to have at least 3 PNC visits. The availability and access to PNC services in rural communities, particularly at the home-based level and male involvement in maternal health care could improve the use of PNC services in rural settings.

These key recommendations could help improve the use of PNC services:

- The Ghana Health Service needs to scale-up the CHPS program to give all community members, particularly pregnant women and nursing mothers in rural communities, the opportunity to access health services at their doorsteps.
- The Ghana Health Service needs to roll out the home-based PNC services, beginning with rural communities where distance from care may be a challenge for women with newborn babies.
- Health professionals could use more innovative approaches to reach out to women in the West Mamprusi district to increase the use of PNC services.
- Health professionals need to educate and engage men in maternal health programs.

## Abbreviations

ANC: Antenatal Care
AOR: Adjusted Odd Ratio
CHOs: Community Health Officers
CHPS: Community-based Health Planning and Services
CI: Confidence Interval
EA: Enumeration Area
HIV: Human Immunodeficiency Virus
MDGs: Millennium Development Goals
NHIS: National Health Insurance Scheme
PHC: Primary Health Care
PNC: Postnatal Care

## References

[1] WHO Trends in Maternal Mortality: 1990 to 2015. WHO. [cited 2016 May 13]. Available from: http://www.who.int/reproductivehealth/publications/monitoring/maternal-mortality-2015/en/.

[2] Warren C, Daly P, Toure L, Mongi P (2006). Opportunities for Africa’s newborns. “Postnatal care.”.

[3] UNDP. Ghana Millennium Development Goals, 2015 Report [Internet]. Accra, Ghana; 2015 [cited 2016 Jul 14]. Available from: http://www.gh.undp.org/content/ghana/en/home/library/poverty/2015-ghana-millennium-development-goals-report.html.

[4] Ronsmans C, Graham WJ (2006). Maternal survival series steering group., maternal mortality: who, when, where, and why. Lancet.

[5] Say L, ChouD Gemmill A, Tunçalp O, Moller A, Daniels J, Gülmezoglu MA, Temmerman M, Alkema L (2014). Global causes of maternal death: a WHO systematic analysis. Lancet Glob Health.

[6] GBD 2013 Mortality and Causes of Death Collaborators (2015). Global, regional, and national age-sex specific all-cause and cause-specific mortality for 240 causes of death, 1990–2013: a systematic analysis for the Global Burden of Disease Study 2013. Lancet.

[7] WHO. WHO Recommendations on Postnatal Care of the Mother and Newborn. Geneva, Switzerland; 2013 [cited 2016 Aug 5]. Available http://www.who.int/maternal_child_adolescent/documents/postnatal-carerecommendations/en/. 24624481

[8] Ghana Health Service. Ghana National Safe Motherhood Service Protocol [Internet]. Accra, Ghana Health Service; 2008 [cited 2016 Apr 7]. Available from: http://mtshohoe.edu.gh/library/index.php?keywords=National+Safe+Motherhood+Service+Protocol&search=search.

[9] Ministry of Health. Ghana National Newborn Health Strategy and Action Plan 2014–2018. 2014. Available from: https://www.ghanahealthservice.org/downloads/Ghana_National_Newborn_Strategy_Final_Version_March_27.pdf.

[10] Ghana Statistical Service (GSS), Ghana Health Service (GHS), and ICF Macro (2015). Ghana Demographic and Health Survey 2014 Key indicators.

[11] Chakraborty N, Islam MA, Chowdhury RS, Bari W (2002). Utilization of postnatal care in Bangladesh: evidence from a longitudinal study. Health Soc Care Community.

[12] Ciceklioglu M, Soyer MT, Öcek ZA (2005). Factors associated with the utilization and content of prenatal care in a western urban district of Turkey. Int J Qual Health Care.

[13] Dhaher E, Mikolajczyk RT, Maxwell AE, Krämer A (2008). Factors associated with lack of postnatal care among Palestinian women: a cross-sectional study of three clinics in the West Bank. BMC Pregnancy Childbirth..

[14] Dhakal S, Chapman GN, Simkhada PP, van Teijlingen ER, Stephens J, Raja AE (2007). Utilisation of postnatal care among rural women in Nepal. BMC Pregnancy Childbirth.

[15] Hordofa MA, Almaw SS, Berhanu MG, Lemiso HB (2015). Postnatal care service utilization and associated factors among women in dembecha district, Northwest Ethiopia. Sci J Public Health..

[16] Hove I, Siziya S, Katito C, Tshimanga M (1999). Prevalence and associated factors for non-utilisation of postnatal care services: population-based study in Kuwadzana Peri-urban area, Zvimba District of Mashonaland West Province. Zimbabwe Afr J Reprod Health.

[17] Khanal V, Adhikari M, Karkee R, Gavidia T (2014). Factors associated with the utilisation of postnatal care services among the mothers of Nepal: analysis of Nepal demographic and health survey 2011. BMC Womens Health.

[18] Mrisho M, Obrist B, Schellenberg JA, Haws RA, Mushi AK, Mshinda H, Schellenberg D (2009). The use of antenatal and postnatal care: perspectives and experiences of women and health care providers in rural southern Tanzania. BMC Pregnancy Childbirth..

[19] Titaley C, Dibley M, Roberts C (2009). Factors associated with non-utilisation of postnatal care services in Indonesia. J Epidemiol Community Health.

[20] Workineh G, Hailu DA (2014). Factors affecting utilization of postnatal care service in Jabitena district, Amhara region, Ethiopia. Sci J Public Health.

[21] UNICEF. Project for Improving Access to Quality Health and Education Services in the Northern and Upper East Regions of Ghana-An Endline Evaluation. Ghana; 2017. Report No.: 9127709

[22] Nyonator FK, Awoonor-Williams JK, Phillips JF, Jones TC, Miller RA (2005). The Ghana community-based health planning and services initiative for scaling up service delivery innovation. Health Policy Plan.

[23] Sakeah E, McCloskey L, Bernstein J, Yeboah-Antwi K, Mills S, Doctor HV (2014). Is there any role for community involvement in the community-based health planning and services skilled delivery program in rural Ghana?. BMC Health Serv Res.

[24] Ghana Health Service. Community-Based Health Planning and Services (CHPS)-Operational policy document. Policy Document No. 20 [Internet]. Ghana; 2005 [cited 2016 Sep 15]. Available from: http://www.moh.gov.gh/wp-content/uploads/2016/02/CHPS-Operational-Policy-2005.pdf.

[25] Ghana Statistical Service. 2010 Population and Housing Census: Summary Report of Final Results. In Accra, Ghana: Ghana Statistical Service; 2012 [cited 2013 Dec 16]. Available: http://www.statsghana.gov.gh/docfiles/2010phc/2010_POPULATION_AND_HOUSING_CENSUS_FINAL_RESULTS.pdf.

[26] Ministry of Local Government. Ghana Districts - A repository of all Local Assemblies in Ghana. 2014. Available from: http://ghanadistricts.com/Home/Reader/6932736-59fd-4a13-be.

[27] Dean AG, Sullivan KM, Soe MM. OpenEpi: Open Source Epidemiologic Statistics for Public Health. 2011 [cited 2013 Dec 19]. Available from: http://www.openepi.com.

[28] Pell M, Meñaca A, Were F, Afrah AN, Chatio S, Manda-Taylor L, Hamel JM, Hodgson A, Tagbor H, Kalilani L, Ouma P, Pool R (2013). Factors affecting antenatal care attendance: results from qualitative studies in Ghana, Kenya and Malawi. PLoS One.

[29] Overboscha GB GB, NNN N-N, Van den Booma GJM GJM, Damnyag L (2004). Determinants of antenatal care use in Ghana. Ournal Afr Econ.

[30] Browne JL, Kayode GA, Arhinful D, Fidder SAJ, Grobbee DE, Klipstein-Grobusch K (2016). Health insurance determines antenatal, delivery and postnatal care utilisation: evidence from the Ghana demographic and health surveillance data. BMJ Open.

[31] Abekah-Nkrumah G, Abor PA (2016). Socioeconomic determinants of use of reproductive health services in Ghana. Health Econ Rev.

[32] Kanté AM, Chung CE, Larsen AM, Exavery A, Tani K, Phillips JF. Factors associated with compliance with the recommended frequency of postnatal care services in three rural districts of Tanzania. BMC Pregnancy Childbirth. 2015;15:341. Available from: https://www.ncbi.nlm.nih.gov/pmc/articles/PMC4687308/. 10.1186/s12884-015-0769-8 PMC4687308 26689723

[33] Abor AP, Abekah-Nkrumah G, Sakyi K, Adjasi KC, Abor J (2011). The socio-economic determinants of maternal health care utilization in Ghana. Int J Soc Econ.

[34] Dahiru T, Oche MO (2015). Determinants of antenatal care, institutional delivery and postnatal care services utilization in Nigeria. Pan Afr Med J.

[35] Tesfahun F, Worku W, Mazengiya F, Kifle M (2014). Knowledge, perception and utilization of postnatal Care of Mothers in Gondar Zuria District, Ethiopia: a cross-sectional study. Matern Child Health J.

[36] Neupane S, Doku DT (2012). Determinants of time of start of prenatal care and number of prenatal care visits during pregnancy among Nepalese women. J Community Health.

[37] Rwabufigiri NB, Mukamurigo J, Thomson RD, Hedt-Gautier LB, Semasaka SJP (2016). Factors associated with postnatal care utilisation in Rwanda: a secondary analysis of 2010 demographic and health survey data. BMC Pregnancy Childbirth..

[38] Sakeah E, McCloskey L, Bernstein J, Yeboah-Antwi K, Mills S, Doctor HV (2014). Can community health officer-midwives effectively integrate skilled birth attendance in the community-based health planning and services program in rural Ghana?. Reprod Health.

[39] Lloyd CB, Gage-Brandon AJ (1993). Women’s role in maintaining households: family welfare and sexual inequality in Ghana. Popul Stud.

[40] Adongo PB, Phillips JF, Kajihara B, Fayorsey C, Debpuur C, Binka FN (1997). Cultural factors constraining the introduction of family planning among the Kassena-Nankana of northern Ghana. Soc Sci med 1982.

[41] Ganle JK, Obeng B, Segbefia AY, Mwinyuri V, Yeboah JY, Baatiema L (2015). How intra-familial decision-making affects women’s access to, and use of maternal healthcare services in Ghana: a qualitative study. BMC Pregnancy Childbirth..

[42] Somé DT, Sombié I, Meda N (2013). How decision for seeking maternal care is made—a qualitative study in two rural medical districts of Burkina Faso. Reprod Health.

[43] Furuta M, Salway S (2006). Women’s position within the household as a determinant of maternal health care use in Nepal. Int Fam Plan Perspect.

[44] Mullany BC, Becker S, Hindin MJ (2007). The impact of including husbands in antenatal health education services on maternal health practices in urban Nepal: results from a randomised controlled trial. Health Educ Res.

